# The environmental genomics of metazoan thermal adaptation

**DOI:** 10.1038/hdy.2014.119

**Published:** 2015-03-04

**Authors:** D Porcelli, R K Butlin, K J Gaston, D Joly, R R Snook

**Affiliations:** 1 Department of Animal and Plant Sciences, University of Sheffield, Sheffield, UK; 2 Sven Lovén Centre—Tjärnö, University of Gothenburg, Strömstad, Sweden; 3 Environment and Sustainability Institute, University of Exeter, Penryn, UK; 4 Laboratoire Evolution, Génomes et Spéciation, CNRS—UPR 9034, Gif sur Yvette, France; 5 Université Paris-Sud, Orsay, France

## Abstract

Continued and accelerating change in the thermal environment places an ever-greater priority on understanding how organisms are going to respond. The paradigm of ‘move, adapt or die', regarding ways in which organisms can respond to environmental stressors, stimulates intense efforts to predict the future of biodiversity. Assuming that extinction is an unpalatable outcome, researchers have focussed attention on how organisms can shift in their distribution to stay in the same thermal conditions or can stay in the same place by adapting to a changing thermal environment. How likely these respective outcomes might be depends on the answer to a fundamental evolutionary question, namely what genetic changes underpin adaptation to the thermal environment. The increasing access to and decreasing costs of next-generation sequencing (NGS) technologies, which can be applied to both model and non-model systems, provide a much-needed tool for understanding thermal adaptation. Here we consider broadly what is already known from non-NGS studies about thermal adaptation, then discuss the benefits and challenges of different NGS methodologies to add to this knowledge base. We then review published NGS genomics and transcriptomics studies of thermal adaptation to heat stress in metazoans and compare these results with previous non-NGS patterns. We conclude by summarising emerging patterns of genetic response and discussing future directions using these increasingly common techniques.

## Introduction

A wide array of observed biological changes in organisms have been attributed to the influence of anthropogenic climate change, including in body size (see, for example, Caruso *et al.*, 2014), phenology (see, for example, Parmesan and Yohe, 2003; Menzel *et al.*, 2006), behaviour (see, for example, Llusia *et al.*, 2013) and local abundance and geographic distribution (see, for example, Gaston, 2003; Chen *et al.*, 2011). Although often confirming predictions rooted in ecological and evolutionary theory, other studies have failed to document such responses, and doubtless many more that have done so have never been published (the so-called ‘file drawer' problem). Indeed, a recent synthesis found that of the 73 mammal species in North America that have been assessed for responses to climate change, only 52% have responded as expected, 7% responded in the opposite direction and the remaining 41% have not responded (McCain and King, 2014). Yet the ability to predict accurately how organisms respond to climate change is of utmost importance.

Climate change encompasses systematic alterations in a variety of abiotic and biotic factors. One abiotic factor, temperature, strongly affects the integrity of proteins and cellular structures and rates of physiological processes, particularly in ectotherms, which represent the majority of metazoans. Given the strong effect of temperature on physiology, adaptations to withstand thermal stress are present among all organisms (for a comparison of thermal tolerances in 2740 terrestrial species, see Araújo *et al.*, 2013). Although some of these adaptations are associated with short-term temperature stress, adaptations related to the thermal environment have an important role in determining the geographic range a species can occupy (see, for example, Bozinovic *et al.,* 2011). Accordingly, much attention has been given to how organisms may respond to the changing thermal environment. The focus by many ecologists has been on documenting field and experimental responses to spatial and temporal climate change at higher levels of biological organisation such as species, communities and ecosystems, and using these observations to predict what form future changes might take. In contrast to ecologists, evolutionary biologists tend to focus on the identification of genes (for example, heat shock genes) and their proteins (for example, heat shock proteins (Hsps)) that are critical in mounting a thermal response, particularly at the lower levels of biological hierarchy (molecules, cells, individuals) in model organisms.

However, improving predictions for the way species will respond to climate change requires a much better understanding of how influences at lower levels of the organisational hierarchy are manifested across higher levels, including non-model species. Macrophysiology attempts to link variation in physiological traits over large geographical and temporal scales to the ecological implications of that variation (Chown and Gaston, 2008). Such linkage needs to include identification of the number and distribution of candidate genes that facilitate thermal responses across landscapes given that physiological traits are determined by interactions between genes that control responses to extracellular and environmental cues about thermal stress.

The advent of relatively inexpensive next-generation sequencing (NGS) methods may provide a tool to begin to examine genomic responses to temperature stress, potentially revealing new understanding of responses to environmental change. One clear advantage of NGS over traditional genetic methods for examining response to temperature stress is that it can be applied to both model (those with a genome sequenced and functionally annotated) and non-model (without a genome sequenced or with no or limited species-specific functional annotation) organisms and it provides a whole-organism snapshot of responsive genes. Moreover, a ‘macro' level approach can be accomplished through comparisons within and among populations and species and across landscape scales (see, for example, Somero, 2012). The use of multiple NGS methods (for example, genome sequencing and transcriptomics) combined with proteomics and metabolomics may ultimately link lower and higher levels of biological organisation. Subsequent functional genetic analysis and estimates of selection could lead to improved predictions about whether and how organisms will respond to a rapidly changing thermal environment.

In this contribution, we discuss the application of NGS technologies to study the adaptation to heat stress. We review the recent literature on metazoan studies using NGS genomics and transcriptomics techniques (hereafter just NGS) to understand adaptive responses to heat. We limit our review to metazoans because they have lower thermal tolerances compared with non-metazoans (see, for example, Dilly *et al.,* 2012), suggesting that they are more vulnerable to changes in the thermal environment. We limit our review to heat stress given nearly all identified NGS studies have been on heat response and a recent review of over 1000 metazoans suggests that heat tolerance is more limited than cold tolerance (Araújo *et al.*, 2013). As our focus is on results from NGS studies, we do not summarise phenotypic and physiological responses to heat stress (but see De Jong and Bochdanovits, 2003; Angiletta, 2009; Bozinovic *et al.,* 2011; Araújo *et al.*, 2013 for such reviews). In addition, although a rich literature exists regarding genes that are associated with environmental response determined either by functional genetic or phenotypic associations, an extensive review of genes previously identified is beyond the remit of this review. We do, however, use a recently collated data set on non-NGS identified stress response genes in *D. melanogaster* to compare with emerging NGS results.

## Thermal adaptation pre-NGS

Adaptation to the thermal environment occurs when selection operates on traits associated with thermal stress response and when such variation is genetically mediated. Within a population, trait variation represents what is available for selection to act on, whereas variation between populations represents the outcome of divergent selection (mitigated by gene flow and genetic drift). Combining studies of both intra- and interpopulation variation in response to the thermal environment can be a powerful framework for determining what facilitates and limits adaptive evolution to temperature. This is particularly true in a widely distributed species where populations experience different environmental temperatures. In species with sufficiently low gene flow, divergent selection pressures between environments can result in local adaptation. Local adaptation refers to genetic differentiation among populations that results in increased mean fitness in the local environment (reviewed by Savolainen *et al.,* 2013). Although there is evidence for local adaptation to environmental temperature gradients in a variety of organisms (see, for example, Bozinovic *et al.,* 2011; Savolainen *et al.,* 2013), it remains a fundamental evolutionary question what genetic changes underpin adaptation generally (Stapley *et al.,* 2010), and here specifically to the thermal environment.

The link between genotype and phenotype is particularly critical in studies of thermal adaptation because understanding what limits adaptation to thermal changes is crucial for predicting species responses (see, for example, Hansen *et al.,* 2012). For example, physiological mechanisms that allow response to extreme thermal stress over a short period, to limit mortality, may be different from those that are initiated during sublethal thermal stress but have longer-term negative impacts on fitness (see, for example, Terblanche *et al.,* 2011). Do the same genes, or same classes of genes, control these physiological responses to different thermal experiences or are different genes involved in different responses? If these are different, then linking such responses with climatic predictions about the frequency of different thermal scenarios needs to be made. Likewise, how populations differ in plastic responses to thermal stress (see, for example, Chevin *et al.,* 2013; Smith *et al.,* 2013) and the extent to which populations have the genetic architecture necessary to keep up with the velocity of change (Comte and Grenouillet, 2013) are important parameters for better predictive ability regardless of the climatic scenario. We explore these questions in our literature review.

A recent review of the evolution of thermal tolerance found that both intra- and interspecific variation in heat tolerance was less than for cold tolerance, suggesting that response to heat stress would be limited (Araújo *et al.*, 2013). While summarising phenotypic thermotolerance of over 1000 metazoans, no genotypic data that could inform about the relevant genes of interest were included. Obviously, this is a big task and, for the majority of the 1000 species, little specific genetic information would be available.

In contrast to this large phenotypic study, much is known about genes associated with phenotypic responses to environmental stress in one organism, *D. melanogaster*. A candidate gene database of stress traits in *D. melanogaster*, CESAR, contains 1307 genes associated with environmental stress response (http://pearg.com/cgdfront/). These associations were determined either by functional genetic analysis of response to heat, cold, starvation and dessication (and subsequently catalogued as such; Supplementary Figure S1) or associated with geographical variation in these physiological traits. This is a large list and understanding the function of these genes, and how they interact with each other, can suggest biological hypotheses about adaptation to the thermal environment. In the CESAR data set, the majority of genes are related to starvation response, with heat response the next largest (Supplementary Figure S1). There is some overlap between the genes in terms of their function. For example, of the 376 genes catalogued as heat responsive, 89 also have annotation for starvation response (Supplementary Figure S1).

Another way to analyse these gene lists is to use gene ontology (GO) analysis, which allows categorisation of the responding genes and determining whether there is enrichment of genes for particular biological processes, molecular functions and cellular components. Such analysis may be a useful approach to identify core biological mechanisms that may be critical in thermal adaptation. We used the CESAR list and performed a GO term analysis using DAVID (Huang da *et al.*, 2009) to identify significant categories of genes associated with biological processes, cellular components and molecular functions associated with those 1307 genes. Few GO terms were statistically significant with a false discovery rate cutoff of 5% in any of these categories (Supplementary Table S1), but for biological processes there were three enriched gene categories, corresponding to 37 unique genes (Supplementary Table S1). As this list was collated from the literature on responses to environmental stress, its unsurprising that these genes were enriched for stress responses, and included several heat shock protein (*hsp*) genes, *Fst* (*frost*) and the Turandot family of immune function genes. Other genes in this list had additional interesting annotated functions including *per* (*period*), associated with circadian clocks; *mth* (*methuselah*) associated with aging; and *rut* (*rutabaga*) associated with learning and memory.

The current literature leaves us with a large gap between what is known generally at the macro level about physiological traits related to thermal stress and the underlying genes and genetic architecture of these responses. Linking these two is where NGS studies could add tremendously to our understanding of thermal adaptation and limits to such responses. Below we discuss the uses and challenges of NGS in the study of thermal adaptation.

## NGS technologies and their value for studying thermal adaptation

NGS methods (here meaning high-throughput sequencing of DNA or RNA) provide information on genetic variation and/or on gene expression within and among populations. This information can be used to address all of the steps that Hansen *et al.* (2012) identify for demonstrating an adaptive response to climate change (their Table 3: existence of genetic variation, link to environmental stress, change over time, signatures of selection, link to a specific environmental variable and ruling out population replacement). However, the evidence will generally remain correlative unless the functional links between genetic changes and adaptive phenotypes can be tested. This is not something that can be achieved with NGS techniques alone. High throughput is the key advance of NGS technologies, allowing wider genomic coverage and resolution than approaches that were developed using other sequencing, genotyping or expression methods. However, it can be challenging to use this potential effectively and there is a particular problem in scaling up numbers of samples. Here we briefly discuss the potential applications of NGS to thermal adaptation, solutions to the sample size problem and the challenges of making the genotype–phenotype connection.

Applications of genomics *sensu stricto* fall into three categories: reference genome sequencing, quantitative trait locus (QTL) mapping or analysis of genetic variation within and among populations. Reference genome sequencing provides the raw material that then can be used in any downstream analysis with other NGS technologies. For QTL mapping, NGS provides genome-wide markers at unprecedented density even in non-model organisms, in which QTLs are determined via either laboratory crosses in which parentage is controlled, pedigree analyses or association analyses. For example, Everett and Seeb (2014) recently mapped more than 3500 markers in the Chinook salmon genome and detected three significant QTL for temperature tolerance. Generating a linkage map is a key step, requiring a controlled cross that can be a limitation is some species, although with many markers a single generation can be sufficient (see, for example, Amores *et al.*, 2011). Further interpretation of QTL is then limited by the mapping resolution and the quality of genome annotation. Association analysis requires a higher density of markers and/or large sample size but can increase resolution. We are not aware of any example of its application to thermal adaptation to date (but see Howard *et al.*, 2014 in which the availability of high-density single-nucleotide polymorphism (SNP) marker sets and annotation provided sufficient information on gene functions to characterise loci in candidate genomic regions for body temperature control under thermal stress in cattle).

Genetic variation within and among natural populations can provide evidence for adaptation through signatures of selection such as high differentiation relative to background (‘outlier' approaches, see for example, Luikart *et al.*, 2003), clinal patterns or correlations with environmental variables (Coop *et al.*, 2010) or reduced variation within populations and high linkage disequilibrium (LD; due to ‘selective sweeps', Pritchard *et al.*, 2010). These approaches certainly benefit from the high density of markers provided by NGS, especially where they can be placed on a physical or linkage map (see, for example, Jones *et al.*, 2012). However, population genomic analyses do suffer from false positives generated through uncertainty about population history (Hermisson, 2009) and genome structure (for example, chromosomal inversions generating LD; Kolaczkowski *et al.*, 2011) and can probably only detect loci under strong selection. Power is increased if replicated comparisons are possible (Jones *et al.,* 2012) and analytical approaches continue to advance, improving the ability to separate effects of selection from confounding factors (see, for example, Duforet-Frebourg *et al.*, 2014). Clinal analyses have been particularly important in the analysis of thermal adaptation (see below). Similar approaches can also be applied to temporal comparisons and outlier approaches underpin the ‘evolve and resequence' strategy (E&R, reviewed by Kofler and Schlötterer, 2014). E&R has been applied successfully to thermal adaptation in *Drosophila* (Tobler *et al.*, 2014), although inversions make the identification of key adaptive loci difficult. As with QTL analysis, the step from identifying a region influenced by selection to a specific target locus for further work on the connection through the phenotype to the environment depends on genomic resolution and the quality of genome annotation.

Analysis of gene expression using NGS data (transcriptomics) provides a different type of insight. It is a major approach in stress physiology to gain insights into regulatory changes that accompany patterns of variation, whether plastic or adaptive (Stapley *et al.*, 2010; Whitehead, 2012 and references therein, but see Feder and Walser, 2005). For organisms used as ecological models and for field studies, NGS transcriptomics involves significant challenges beginning with the preservation of samples and extraction of good-quality RNA (Gayral *et al.,* 2011) and continuing with the construction of a reliable *de novo* transcriptome assembly or mapping of reads to a heterologous reference (Cahais *et al.,* 2012). Field studies may also require high biological and technical replication to provide reliable tests of hypotheses. Interpretation again depends on the quality of annotation of the reference. Nevertheless, expression-based approaches have great potential to reveal interacting networks of genes that respond to stress and so to provide insights that complement genomic approaches (Ayroles *et al.*, 2009).

Changes in the gene expression associated with thermal adaptation occur on a wide range of timescales, including long-lasting effects of early-life exposures, maternal effects and transgenerational effects. These changes may be regulated epigenetically, that is, by inherited modifications of the DNA or chromatin other than base sequence changes (such as DNA methylation, covalent histone modifications and DNA silencing by non-coding RNAs). Epigenetic modifications regulate genome expression and chromatin conformation and have a crucial role in response to heat exposure during early development in a number of vertebrate species that strongly impact on adult phenotype. Such modifications can influence population demography and dynamics, particularly in the context of climatic change, and DNA methylation can be detected by NGS methods (Frésard *et al.*, 2013). However, studies combining technologies to identify epigenomic signatures with phenotypic variation are rare and limited by similar issues of NGS already discussed.

The ideal experiment, genomic or transcriptomic, will typically require maximum coverage and high replication. As costs decline, trade-offs are becoming less severe but strategies are still needed to maximise the cost effectiveness of NGS studies. Available strategies include reduced representation, either ‘randomly' or by targeting, pooling or low-depth methods. RAD-seq (Baird *et al.,* 2008) and related genotyping by sequencing methods (see, for example, Parchman *et al.,* 2012) sample the genome using restriction enzymes to provide repeatable sample points. They provide high marker densities that are valuable for mapping studies but the reliance on restriction sites can be problematic for population studies, especially where heterozygosity is high (Gautier *et al.*, 2013) or where comparisons are made across divergent populations or species (Arnold *et al.*, 2013). Targeted capture sequencing relies on prior knowledge of the genome but can provide a directed sample, for example focused on a candidate region (Nadeau *et al.*, 2012) or gene family (Smadja *et al.*, 2012). This approach can also be applied to RNA, where it is capable of accurately representing expression levels for a targeted subset of the transcriptome (see, for example, Levin *et al.*, 2009). Combined with multiplexing of indexed samples, these genome sampling approaches allow many more individuals or treatments to be studied for the same sequencing cost but they inevitably miss some patterns that might be detected with sequencing of whole genomes or transcriptomes.

Sequencing of whole genomes for many individuals remains prohibitively expensive for most laboratories, although this may not be true for long. Two contrasting solutions have been proposed: to index individuals and sequence at very low coverage (1 × ; Buerkle and Gompert, 2013) or to pool DNA from many individuals without indexing (Futschik and Schlötterer, 2010). Both approaches sacrifice information at the individual level for the sake of cost-effective estimation of population-level parameters (for individual SNPs, much information on LD is lost; Cutler and Jensen, 2010). Both methods also have to make assumptions, for example, that copy-number variation is low. The low-coverage approach has not yet been applied to thermal adaptation but pool-seq has become a valuable part of the E&R approach (Schou *et al.*, 2014; Schlötterer *et al.*, this volume) and applied to thermal adaptation (Fabian *et al.,* 2012). Both approaches have great potential for the study of natural populations because a high number of individuals can be analysed.

NGS methods can identify gene regions associated with thermal adaptation, transcripts whose expression levels change in response to thermal stress and epigenetic modifications that influence gene expression. However, they must be complemented with other approaches for a full understanding of adaptive responses. In principle, other ‘omic technologies have great potential for filling in the links between genes, phenotypes and fitness. Proteomics and proteogenomics can provide information on protein levels and functional modifications that are not predicted by gene expression (Dilly *et al.,* 2012; Somero, 2012). During stress adaptation and drastic or sudden temperature changes, rapid gene expression changes may underlie production of various proteins (including molecular chaperone, protease and other classes of proteins) involved in the protection of cells against damage (Leach *et al.*, 2012). However, protein production, post-translational modifications and protein–protein interactions can change independently of gene expression and so proteomic approaches can complement RNA-based methods to identify physiological or metabolic pathways involved in temperature adaptation (Ibarz *et al.,* 2010; Tomankek and Zuzow, 2010; Serafini *et al.,* 2011; Dilly *et al.,* 2012; Fields *et al.,* 2012). Approaches investigating the proteomic reaction norm of organisms also help to predict their tolerance limits with respect to global warming and, combined with epigenetics, would provide information on any transgenerational inheritance of acquired resistance and resilience of cellular phenotypes (Silvestre *et al.,* 2012). The effects of allelic substitutions, expression changes or protein interactions may be further understood using metabolomics approaches, which seek to quantify and identify complete sets of metabolites using chromatography and mass spectrometry, taking advantage of NGS information (see, for example, Koek *et al.*, 2011).

Combining these approaches has a great potential for the future but will be highly challenging, especially in non-model organisms. It requires a high level of genome and transcriptome information as well as understanding of metabolic pathways that might not transfer reliably from the most closely related model species to the study species. Even when the combined approaches point to promising candidate genes, experimental tests using transformation, directed mutagenesis or expression knockdowns are needed. These techniques are starting to be available for a wider range of organisms (Mohr *et al.,* 2014) and will, no doubt, soon be applied to the study of thermal adaptation.

Given the potential of such ‘omics technologies, we next review the literature on studies employing NGS approaches to study thermal adaptation to heat in metazoans, and determine whether common signatures of putative thermal adaptation are emerging. Because many NGS technologies are only newly being applied to the question of thermal adaptation, we focus on those technologies that have been used the most in thermal adaptation: population genomics and transcriptomics.

## Literature review

### Identifying studies

To identify relevant population genomics and differential gene expression studies, we queried the ‘Web of Science' repository (URL: http://thomsonreuters.com/thomson-reuters-web-of-science/) until 28 February 2014 using combinations of two key phrases. Each query pair was of the form *a*_*i*_*, b*_*j*_, where *a*_*i*_ ɛ A={‘Next-generation sequencing', ‘RNA-seq', ‘RAD-seq', ‘DNA-seq', ‘Pool-seq', ‘Illumina', ‘Roche 454', ‘SOLiD'} and *b*_*j*_ ɛ B={‘Climate', ‘Thermal adaptation', ‘Heat stress'}. Technology terms were used rather than, for example, ‘transcriptomics' to avoid studies that were not relevant (for example, microarray results). All possible *a*_*i*_ AND *b*_*j*_ combinations were used in our search and, after removing studies covering plant, bacterial or archaeal species, a set of 32 publications, centred exclusively on 27 different metazoan species, was used for subsequent analysis (Table 1). Although this approach should have identified the great majority of relevant papers, we cannot claim that our coverage is exhaustive.

### General patterns

**Figure 1:**
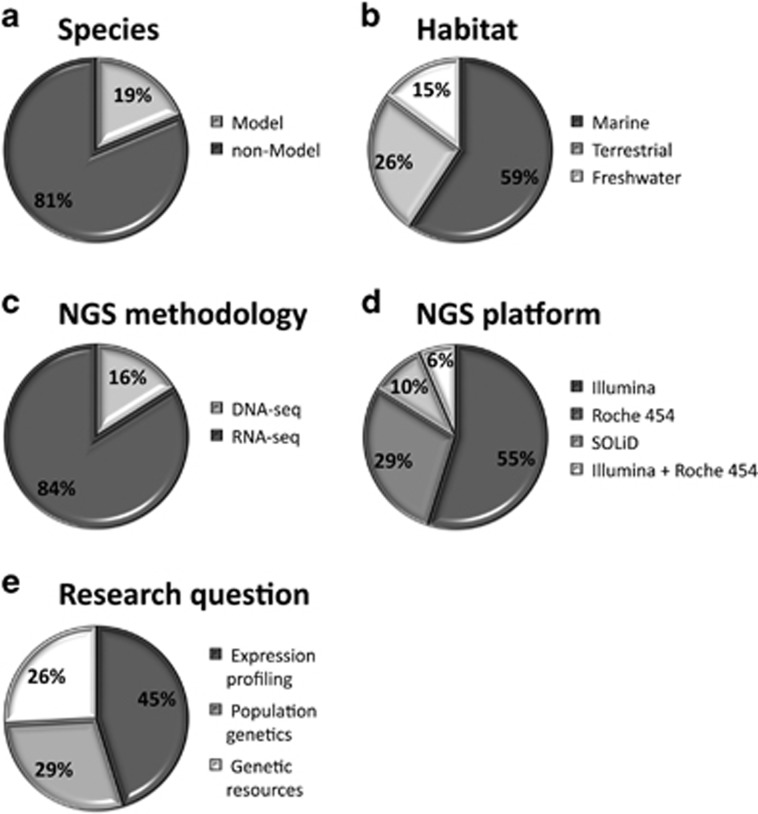
Main features of thermal adaptation studies employing NGS technologies. (**a**) Percentage of studies on model and non-model species; (**b**) habitat of the investigated species; (**c**) NGS methodologies used; (**d**) NGS platforms employed; (**e**) research question addressed (NB: ‘Genetic resources' denote publications that mainly focus on providing transcriptome characterisation and detecting microsatellites and/or SNPs for future applications). See Table 1 for specific studies.

Figure 1 summarises whether studies used NGS for model or non-model species (Figure 1a), the habitat of those species (Figure 1b), the NGS methodology (Figure 1c) and platform (Figure 1d) employed, and the research question addressed (Figure 1e). Confirming that NGS is a useful tool for studying non-model organisms, we find that 81% of the identified studies are on organisms that previously had few genomic resources. Anecdotally, it appears that studies on model organisms are continuing to use microarray-based methodology, although this is likely to change rapidly given that NGS technologies are becoming less expensive. The majority of study species live in marine habitats. Research also fell into five general types of study (Table 1): (1) observational studies that considered genetic patterns across large geographical scales deriving from either laboratory housed or wild populations, (2) experimental evolution in which populations were subjected to thermal selection and genetic responses subsequently surveyed; such studies can also take an E&R approach in which ‘before-' and ‘after-selection' samples are sequenced (see Schlötterer *et al.*, this issue), (3) laboratory heat stress experiments in which a population is subjected to rapid, but short-term, temperature increase, (4) common garden experiments in which populations adapted to different thermal environments (for example, along a species distribution gradient) are subjected to a common temperature and (5) studies focused on the generation of genetic references (for example, SNP markers) and reference transcriptomes. Approach (3) can identify plastic responses to thermal stress, whereas the other techniques can identify signatures of thermal adaptation. Although the majority of these approaches can be used in the wild, most have only been performed in the laboratory.

Among NGS technologies, we found a bias towards the Illumina platform, in particular RNA-seq. This is in contrast to a previous survey on the rising use of NGS for ecological studies which showed a prevalence of the Roche 454 platform (Ekblom and Galindo, 2011) and likely reflects shifts in cost and sequence read length. RNA-seq may be the preferred platform given that this transcriptomic approach provides one data set that generates a reasonable genetic reference and can determine both differential gene expression and sequence polymorphisms. Of the 26 studies on non-model organisms, 24 provide *de novo* transcriptome assemblies. The construction of a high-quality transcriptome is clearly critical for accurate downstream analyses. However, despite its popularity, the RNA-seq Illumina platform generates transcriptomes that are generally oversized (that is, consisting of a very high number of contigs) even when paired end sequences are used for the assembly (see Supplementary Information and Supplementary Figure S2). This oversized transcriptome problem has been noted before (see, for example, Klassen and Currie, 2012) and may generate an overestimation of the number of genes that are linked to thermal adaptation, increasing the number of false positives.

### Population genomics and sequence polymorphisms

A major objective in combining NGS data with a population genetic approach to thermal adaptation is to identify putative adaptive genes and other genetic modifications that may have been influenced by positive selection along a thermal gradient. Of the 32 studies, 10 were focussed on a population genetics approach (Figure 1e; Table 1). The NGS methodologies used hinged on whether an existing genome was available. Population genetic questions on model species (*n*=4; *Drosophila melanogaster*; Table 1) were approached using Illumina DNA-seq technology, which provides information on both exonic and non-exonic genomic regions. In contrast, either RNA-seq or RAD sequencing or both was used on non-model organisms (Table 1); with RNA-seq, information on coding regions and untranslated regions (UTRs) will be identified, whereas with RAD sequencing whole-genome scanning can identify both coding and non-coding loci of interest but these cannot be associated with a genomic map in the absence of a reference genome. Studies focused on: (i) identifying genetic loci that are targets of thermal selection; (ii) observing the nature of the genetic modifications, such as synonymous or non-synonymous changes, alterations in regulatory sequences and chromosomal inversions and (iii) identifying enriched classes of genes associated with spatially varying thermal selection. Below we summarise these studies, differentiating between model and non-model organisms.

#### Model systems

Recent studies using *Drosophila melanogaster* illustrate the efficacy of combining NGS DNA-pool data with a high-quality genomic resource to elucidate thermal adaptation. We found three observational investigations focusing on genetic differentiation in natural *D. melanogaster* populations along Australian and North American latitudinal clines, associated with spatially varying thermal selection (Kolaczkowski *et al.*, 2011; Fabian *et al.*, 2012; Reinhardt *et al.*, 2014). A previous study used array technology to study these same clines (Turner *et al.*, 2008), but the use of NGS allows discovery of genetic differentiation at the nucleotide level. These studies sequenced the genome of pooled individuals from populations from either the two ends of the Australian cline (Kolaczkowski *et al.*, 2011) or the two ends and a mid-cline population from North America (Fabian *et al.,* 2012) or the same four populations from the two ends of both continents at the same time (Reinhardt *et al.*, 2014). This last study is of particular interest as it focuses on parallel genomic responses to clinally varying selection and summarises the similarity in the findings with the previous studies. Interestingly, the majority (64%) of SNPs segregating on both clines were found to be convergent. Parallel genetic differentiation at 807 candidate genes was identified using *F*_ST_ outlier window analysis (personal communication from the corresponding author; Reinhardt *et al.*, 2014). Moreover, GO analysis showed the genes responding in parallel to be significantly enriched for many biological processes, particularly related to regulation of transcription, and various processes of development and morphogenesis (see text and supporting file from Reinhardt *et al.,* 2014). Many of these GO terms agree with previous observations for the individual clines (Kolaczkowski *et al.* 2011; Fabian *et al.* 2012).

Another study using *D. melanogaster* employed experimental evolution and the E&R approach in which replicated and sequenced laboratory adapted populations (Orozco-terWengel *et al.*, 2012) were subjected to either a hot or a cold environment for 15 generations and then resequenced (Tobler *et al.,* 2014). This study found many genes with outlier SNPs in both the hot and cold treatments. To examine these candidates, the researchers tested the association between thermal response during experimental evolution and previous studies identifying candidate genes using the CESAR data set, curated only for genes putatively associated with thermal adaptation (*n*=340). This comparison identified 47 genes that had outlier SNPs that responded in the predicted direction during experimental evolution (that is, heat or cold stress genes for the hot or cold environment, respectively; Tobler *et al.*, 2014).

The clinal study and the experimental evolution study found many SNPs associated with variation in the thermal environment. However, the genomic distribution of these genes may not be independent, that is, there may be physical linkage among them. The cosmopolitan chromosomal inversion polymorphism, *In(3R)Payne*, occurs in both clines, varies in frequency latitudinally and is thought to be important in both genetic and phenotypic differentiation along the clines (Rako *et al.,* 2006). But LD associated with chromosomal inversions may also generate false positives for the number of candidate genes (Kennington *et al.*, 2006). Thus, the numbers of candidate genes found in both studies may be overestimated.

**Figure 2:**
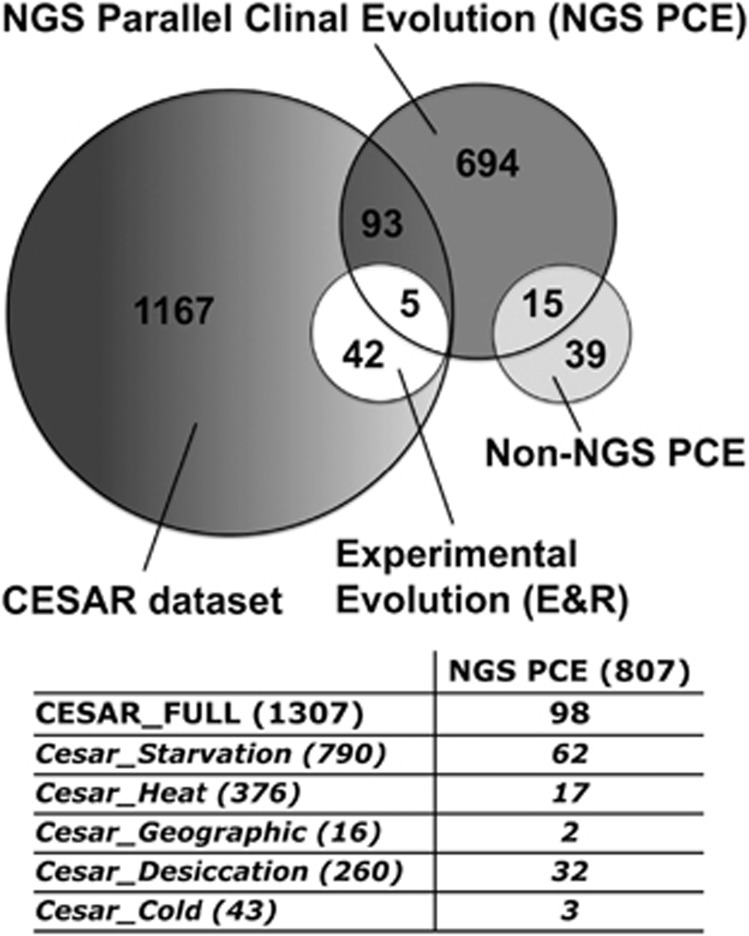
Venn diagram of the overlaps between adaptive and stress responsive *D. melanogaster* genes from Reinhardt *et al.*, 2014 (NGS Parallel Clinal Evolution, NGS PCE), Tobler *et al.*, 2014 (Experimental Evolution, E&R), the CESAR data set and Turner *et al.*, 2008 (non-NGS PCE (non-NGS Parallel Clinal Evolution)). Further details of the overlap between NGS PCE genes and the CESAR functional classes are tabulated below the diagram; the number of genes belonging to each class is reported in parenthesis.

Here we combine results from the clinal (Reinhardt *et al.*, 2014) and experimental evolution (Tobler *et al.*, 2014) studies by examining the overlap between the 807 candidate genes identified from the natural parallel clinal study (Reinhardt *et al.*, 2014), the 47 genes identified from the experimental evolution study (Tobler *et al.*, 2014) and the full CESAR data set (*n*=1307 genes). We found that only five genes overlap between the clinal and experimental evolution studies and 98 between the clinal study and the CESAR list (Figure 2). Of the five genes that co-occur between the clines and the experimental study, we found that two of these genes map close to the chromosomal inversion breakpoints of *In(3R)Payne*, whereas one localises within the inversion itself (Supplementary Information). Two of these are identified as cold tolerance genes (*dpp*, *retn*), two as heat tolerant (*asp*, *l(3)L1231*) and one as both heat and starvation tolerant (*CG6733*).

Using DAVID, we identified GO terms associated with the 98 shared genes between the clinal study and the full CESAR data set. We found several enriched GO terms, with the top scoring ones particularly related to biological processes of neuron differentiation and gamete generation, although the significance of those was lost after correction for multiple tests (see Supplementary Table S2).

#### Non-model systems

As in our analysis for model systems above, we examined the patterns of gene responses for thermal adaptation in non-model systems. We found six thermal adaptation studies on non-model systems (Table 1) that take a population genomics approach; unlike the *D. melanogaster* studies, they did not focus on the nature of the genetic differentiation (likely due to having only a transcriptome resource) but, instead, focused on identifying outlier SNPs and describing their association with gene function categories. GO analysis depends on having high-quality genome annotations so one issue with combining information across disparate non-model organisms is a potential bias in the results. Therefore, to limit any bias, we only analysed studies on related organisms (that is, the three molluscs; Table 1) and provide a summary of representative GO terms across these three molluscans.

The most frequent gene class emerging as consistently under selection, determined via SNP outlier analyses, was related to energy metabolism (two studies out of three; Pante *et al.*, 2012; De Wit and Palumbi, 2013). This class of genes was also observed to have a role in thermal adaptation in the coral *Acropora palmata* (Polato *et al.*, 2011), wheras Lemay *et al.* (2013) discovered two distinct haplotypes within the mitochondrially encoded NADH dehydrogenase subunit 5 associated with an elevation in the American pika, *Ochotona princeps*. NADH dehydrogenase is directly associated with energy metabolism as it is the first complex of the mitochondrial respiratory chain. Energy metabolism is strictly linked to the well-known role of mitochondria in thermal adaptation by their capacity in providing metabolic energy or operating thermoregulation (Keller and Seehausen, 2012).

### Sequence response comparisons between model and non-model systems

We searched for energy metabolism genes associated with thermal responses in the *D. melanogaster* studies reviewed above. However, although energy metabolism was not a significantly enriched GO term for any of the *D. melanogaster* studies, a large number of outliers in the Australian cline mapped onto 3′ UTRs, several of which were involved in energy metabolism (see Supplementary Table S2 in Kolaczkowski *et al.,* 2011 for the top genes showing 3′ UTR differentiation). We also looked at the CESAR data set to determine whether any energy metabolism genes were present and found nine genes; seven genes associated with oxidative phosphorylation (~10% of genes associated with this process) and two associated with the Krebs cycle (see Supplementary Information). These nine genes were not present in the 807 differentiating genes identified in the clinal study.

### Gene expression profiling

RNA-based NGS technologies, especially short-read deep sequencing, are revolutionising approaches to gene expression studies, from a single cell (Deng *et al.*, 2014) to non-model species or community metatranscriptomics (Jorth *et al.*, 2014). Given the benefits and versatility of RNA-seq compared with other gene expression methodologies (for example, microarrays; Mutz *et al.,* 2013), RNA-seq is expected to become the predominant tool for transcriptome analysis, a trend already evident in the most recent studies on genetics of thermal adaptation (Figure 1). Seven studies provided transcriptomic resources without any subsequent analysis but we identified 15 studies that provide 29 comparisons of gene expression in 11 species (Table 1). Of the 29 comparisons, 20 compared the gene expression between a control (benign) and a stressful heat condition within a given population and the remaining nine employed common garden designs, examining interpopulation differential gene expression under fixed thermal regimes (Table 1). Two comparisons were on the model organism, *Strongylocentrotus purpuratus*, and the remainder were on non-model organisms (Table 1). Some studies used whole-body expression data, whereas others used specific or multiple tissues (Table 1).

To examine whether global patterns of differential gene expression can be discerned from the current studies, we first examined whether the number of differentially expressed genes depended on the extent of the temperature shift, the shift duration or the product of these two, using linear regression for each tissue. We tested these relationships because a study on the coral, *Acropora millepora*, reported that longer stress periods resulted in a greater number of genes being differentially expressed (441 vs 75 genes when stressed for 120 or 4 h, respectively; Meyer *et al.,* 2011). We did not find any significant relationships for any variable among the different tissue types (Supplementary Figure S3a), although this may be due to the small number of studies for each tissue type. When considering all tissue types together, we found a positive correlation between the number of differentially expressed genes and the duration of the temperature shift (*r*=0.48, *P*=0.02313, Supplementary Figure S3b), although this is driven by three data points representing relatively large numbers of differentially expressed genes in heat-stressed tissues of *Trematomus bernacchii* (Huth and Place, 2013; Supplementary Figure S3c; see also Supplementary Table S3). Although the duration of the temperature shift in this species was not the longest, it was still quite long and this species is an extremely stenothermal icefish; both factors may contribute to this anomaly.

**Figure 3:**
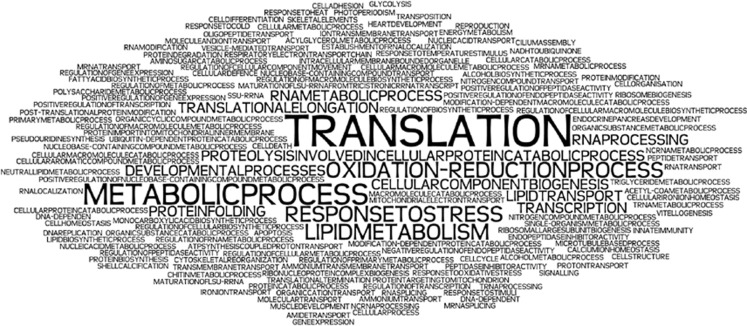
Tag word cloud for GO terms of biological processes associated with differential gene expression studies.

We also summarised enriched GO terms to identify any global patterns of evolutionary response to thermal environments. As we did not have access to the raw data for these 15 studies, we collated each RNA-seq study's list of enriched GO terms and depicted the relative frequency of each term occurring across the studies, using Wordle software (http://www.wordle.net;Figure 3; Table 2). For biological processes, terms related to protein translation were the most frequently identified among all the studies, indicating a possible fundamental role for genes involved in translation (for example, genes encoding ribosomal subunits) during responses to changing temperatures. Metabolic processes, oxidation–reduction processes, response to stress and lipid metabolism were also highly represented. For molecular function, ATP binding and structural constituents of ribosomes were the most frequently identified and for cellular components 50% of the studies found ribosomes as significantly enriched (Table 2).

With respect to plastic vs adaptive responses, six differential gene expression studies took an adaptive common garden approach and 12 studies examined plastic responses by comparing a population's response with heat stress (Table 1). Again, here we examine common reported biological processes GO terms and find that for the adaptive studies, only translation appears more than once (Liu *et al.,* 2013; Pespeni *et al.,* 2013). Because studies examining plastic responses predominate, just analysing those studies for GO term similarity does not change much compared with the analysis including all studies (Figure 3; Table 2). However, if only plastic studies are taken into account, translation and metabolic processes have the same occurrence (33% of the studies).

### Expression and sequence evolution of *hsp* genes

The increasing number of studies on thermal adaptation using NGS allows closer examination of particular classes of genes that are known to be important in thermal responses, for example, heat shock genes (for review see Sørensen *et al.*, 2003). Although much is known about their expression in individual studies and their potential role as capacitors of evolutionary change (Rohner *et al.,* 2013), here we examined the global patterns of response in both differential gene expression and genetic differentiation to determine whether there are common patterns of response.

In 10 out of 15 studies in which a single population was subjected to a temperature shift and then studied for changes in gene expression, members of both the Hsp70 and Hsp90 gene families were upregulated upon heat stress (Meyer *et al.*, 2011; Runcie *et al.*, 2012; Tan *et al.*, 2012; Clark *et al.*, 2013; Huth and Place, 2013; Liu *et al.*, 2013; Narum *et al.*, 2013; Newton *et al.*, 2013; Olsvik *et al.*, 2013; Smith *et al.*, 2013). However, in line with previously observed patterns (Sørensen *et al.,* 2003), *hsp70* and *hsp90* were also found to be downregulated or constant in longer-duration stress (Meyer *et al.*, 2011; Kenkel *et al.*, 2013; Narum *et al.*, 2013). In the common garden experiments, genes belonging to Hsp70, Hsp90 and Hsp47 families showed higher constitutive gene expression levels in more thermally tolerant populations (Schoville *et al.*, 2012; Tan *et al.*, 2012; Barshis *et al.*, 2013; Liu *et al.*, 2013; Narum *et al.* 2013; Newton *et al.* 2013).

The majority of studies we reviewed did not examine sequence evolution of *hsp* genes. However, three studies did find evidence of sequence evolution. These were the 3′ UTR of *hsp47* in *Oncorhynchus mykiss* (Narum *et al.,* 2013), the 3′ UTR of *hsp60B* in *Drosophila melanogaster* (Kolaczkowski *et al.,* 2011) and the putative coding sequences of *hsp68* and *hsp70* in *Nucella lapillus* (Chu *et al.,* 2014). In addition, given that heat shock protein genes are well described, NGS techniques can be useful in understanding the evolution and evolvability of these genes. For example, duplication within the heat shock protein genes may represent a key feature in adaptive genomic processes (Clark *et al.*, 2011; Zhang *et al.*, 2012; Wei *et al.*, 2013). Expansion of Hsp gene families was found in three NGS studies; 11 *hsp70* genes were identified in *Liposcelis entomophila* (psocid; Wei *et al.,* 2013), 9 in *Euphausia superba* (antarctic krill; Clark *et al.,* 2011) and at least 10 copies in *Tigriopus californicus* (copepod; Schoville *et al.*, 2012). Subsequent functional analyses must be undertaken to define specific roles of these genes.

Such changes in *hsp* gene expression (higher expression in heat tolerant populations) and sequence evolution in 3′ UTRs are associated with the evolution of regulatory sequences. These hsp findings corroborate earlier individual studies of *hsp* gene regulation evolution; for example, in *Drosophila*, insertions of transposable elements in the proximal promoters of *hsp* genes are positively selected (Walser *et al.*, 2006) and can change the regulation of the gene (for an example see Wurmser *et al.*, 2013). Another previous example corroborating *hsp* gene regulation evolution in response to the thermal environment is that in some species (for example, Antarctic fish) *hsp70* is now constitutively, rather than inducibly, expressed (Place and Hofmann, 2004).

## Conclusion

We have asked how NGS can add to understanding thermal adaptation from the consideration of whether the same genes, or same classes of genes, control physiological responses to different thermal experiences or whether different genes were involved in different responses, the genetic architecture of such responses and the nature of plastic vs adaptive responses. Below, we summarise our literature analysis and provide some comments on future directions for this nascent research area.

### Emerging patterns

Some intriguing patterns are emerging from the currently small number of studies employing NGS technologies on thermal adaptation in metazoans. NGS genomics studies have shown how specific classes of genes may be targets of selection during adaptation to different thermal environments and have identified some individual candidate loci. Clinal studies of *D. melanogaster* have found strong evidence that adaptive genes are those involved in transcription regulation, development and morphogenesis. Transcriptomic approaches in non-model organisms also reveal some common patterns, emphasising protein translation above all. Strikingly, by providing both genomic and transcriptomic information, these data point to the importance of the gene expression process, that is, from regulation of gene transcription to mRNA translation, in thermal adaptation. This NGS pattern is also found when examining the previously well-studied heat shock proteins. Such information can provide a valuable starting point for subsequent downstream functional analyses, and for NGS studies in other organisms, for understanding how organisms respond to changing thermal conditions.

Other emerging patterns are perhaps not so positive. Although genes involved in energy metabolism are candidate targets for non-model species, no genes in this category were found for the studies on *D. melanogaster* (although outliers mapping onto 3′ UTRs in the Australian *D. melanogaster* cline showed non-significant enrichment for several involved in energy metabolism; Kolaczkowski *et al.,* 2011). Such a conclusion remains cautious, however, given a variety of factors, such as a limited number of species to compare and poor gene annotation in the non-model organisms. Nevertheless, it may be beneficial in future studies to consider genes involved in energy metabolism as good candidates for thermal response, as has been shown in non-NGS studies of other organisms (see, for example, Hancock *et al.*, 2008).

We also found a general lack of overlap between different types of thermal studies, using the same model organism for which there is much genetic information on environmental stress response. Although substantial parallel genetic differentiation was seen between North American and Australian *D. melanogaster* clines (Reinhardt *et al.*, 2014), the overlap of these results compared with an evolve and resequence approach (Tobler *et al.*, 2014) was minimal (<1%). This low overlap is partially due to the E&R study restricting gene analysis to the CESAR data set related to heat or cold stress response only, whereas the clinal study did not use the CESAR data set as a filter. The overlap is also limited because the clinal study shows response for only 20 heat and cold genes, out of over 400 potential targets, from the CESAR list (Figure 2).

NGS techniques provide more fine-scale information on sequence evolution than non-NGS methods. To that end, it is unsurprising that more genes (807) were found to be differentiating in parallel across the North American and Australian clines using NGS than a previous study on the same populations using non-NGS methods (54 genes; Turner *et al.*, 2008). However, of those previously identified genes, only 15 overlapped with the NGS identified genes and none overlapped with the E&R study (Tobler *et al.*, 2014; Figure 2). In addition to technological advances, the populations had also spent more time in laboratory culture between studies.

A tentative conclusion can be made about emerging patterns. When considering the evolution of gene sequences, patterns of similarity appear when using general gene categories (for example, GO analysis). Disparity among studies, even from the same populations, occurs when considering the exact genes that are responding. With respect to gene evolution, we only collated GO terms (and not the actual genes) and found similar response across studies.

### Genetic architecture of thermal response

The genetic architecture of genes responding to thermal stress is critical in determining whether populations can keep up with the velocity of environmental change (Comte and Grenouillet, 2013). Most NGS studies have yet to consider genetic architecture, with the exception of chromosomal inversions. Chromosomal inversions can protect locally adapted genes from homogenising effects of gene flow, but can also result in false positives due to LD. Of the five overlapping genes between the *D. melanogaster* E&R (Tobler *et al.*, 2014) and clinal (Reinhardt *et al.*, 2014) studies, we found that three were associated with the clinally varying *In(3R)Payne*. Whether these are independent targets of selection remains to be determined. Additional NGS techniques and functional genetics can address this question.

Although remaining locally adapted can allow population persistence, whether such microevolution mitigates or aggravates future responses to climate change is debated (see, for example, Kopp and Matuszewski, 2014). Understanding the underlying genetic architecture of thermal responses across populations and species can provide critical information on this debate given that genetic correlations between traits and spatial distribution of genetic variance (Lavergne *et al.,* 2010) may limit local adaptation to thermal challenges. Models of adaptation to either continuously varying environmental optima (Kirkpatrick and Barton, 2006; Bridle *et al.,* 2010) or local adaptation to two contrasting environments (Yeaman and Whitlock, 2011) vary in their predictions about clines of allelic frequencies and whether evolution tends (or not) towards few genes of large effect. The clinal work looking at parallel differentiation in *D. melanogaster* cannot test for such patterns given that only the ends of the clines were examined. Predictions from local adaptation are also sensitive to departures from additivity such as epistasis, or interactions among traits, due to linkage or pleiotropy; as multiple traits must commonly be involved in thermal adaptation, these complexities are important. For example, as shown in our analysis of the CESAR data set, some genes are pleiotropic. Setting studies of thermal adaptation in a genetic architecture framework would be valuable for more detailed understanding of capacity for thermal responses.

### Future experimental designs

‘Omics data have the potential to elucidate the network of genetic, regulatory and metabolic responses to thermal environments from cellular to evolutionary analysis. Although there is much to be gained by integrating these technologies, no published study has yet combined, for example, RNA-seq with either proteomics or metabolomics. And, as stated earlier, genes identified as responding in expression or allele frequency, subsequently requires investigation of the functions of putative candidate genes, genetic variation for the relevant traits within and among populations, the intensity of selection and links between adaptive responses and population dynamics that underlie population persistence. It is no surprise that, given the infancy of NGS, this necessary integration has yet to occur.

A wide variety of experimental designs can be employed to address thermal responses, distinguishing the effects of adaptation and plasticity (Merilä and Hendry, 2014). Here we found six common garden studies examining adaptive gene expression response to heat stress and 12 examining plastic responses. Overall, genes associated with translation appear in many of these studies irrespective of the design. Although this is an interesting pattern, it demands that much more information can be gained by coupling proteomic analyses to these studies.

All the reviewed studies have been performed in the laboratory or on two to three populations (for the one exception, see Chu *et al.,* 2014). Patterns encompassing large geographical scales (or shorter spatial scales in relation to altitude or depth) and across both longer (for example, seasons) or shorter (for example, heat waves) timescales have not yet been considered either in the laboratory or in the wild. Experimental evolution and E&R laboratory designs can provide critical information on the most likely genomic and transcriptomic targets of selection but the ecological relevance of the response is not yet clear (see, for example, Terblanche *et al.,* 2011) and the extent to which long-term laboratory responses, usually to change in only one abiotic factor, are reflected in natural populations is a critical area for development. Although field-based studies may directly reflect ecological relevance, they cannot control for non-genetic effects, which may impact expression, proteomic and metabolomic analyses. Nevertheless, it is important to tackle gene expression under field conditions: the many factors that complicate expectations are precisely those that matter for adaptation to real conditions. Combining studies utilising both natural populations and controlled laboratory environments would shed light on the relevant ecological patterns that need to be replicated in the laboratory to further our understanding of thermal adaptation. Given the rapidly growing availability and decreasing costs of NGS technology, there is much scope for a wide variety of future studies to elucidate the genetic architecture of, and the potential for, thermal adaptation regardless of the future climatic scenario.

## Data archiving

There were no data to deposit.

## Figures and Tables

**Table 1:** The model or non-model species investigated (Class), organised by phylum, their habitat, the NGS platform and methodology, applications (research question) and tissues studied using NGS techniques in thermal adaptation references

*Species*	*Habitat*	*Platform*	*Methodology*	*Research question*						*Tissue*	*Type of study*	*References*
				*Transcriptome characterisation*	*SNPs finding*	*Microsat finding*	*Population genetic*	*DGE profiling*	*GO terms and candidate gene finding*			
*Model*												
Arthropoda												
*Drosophila melanogaster* (Insecta)	Terrestrial	Illumina	DNA-seq		x		x		x	Whole body (adults)	Observational	Kolaczkowski *et al.*, 2011
		Illumina	DNA-seq		x		x		x	Whole body (adults)	Observational	Fabian *et al.*, 2012
		Illumina	DNA-seq		x		x		x	Whole body (adults)	Experimental evolution	Tobler *et al.*, 2014
		Illumina	DNA-seq		x		x		x	Whole body (adults)	Observational	Reinhardt *et al.*, 2014
Echinodermata												
* Strongylocentrotus purpuratus* (Echinoidea)	Marine	SOLiD, Illumina	RNA-seq					x	x	Embryo	Heat stress	Runcie *et al.*, 2012
			RNA-seq					x	x	Tube foot	Common garden	Pespeni *et al.*, 2013
												
*Non-model*												
Annelida												
* Alvinella pompejana* (Polychaeta)	Marine	454, Illumina	RNA-seq	x						Whole body	Genetic reference	Holder *et al.*, 2013
Arthropoda												
* Erynnis propertius* (Insecta)	Terrestrial	454	RNA-seq	x	x					Whole body (larvae)	Genetic reference	O'Neil *et al.*, 2010
* Euphausia superb* (Malacostraca)	Marine	454	RNA-seq	x	x	x				Whole body	Genetic reference	Clark *et al.*, 2011
* Liposcelis entomophila* (Insecta)	Terrestrial	Illumina	RNA-seq	x	x	x				Whole body	Genetic reference	Wei *et al.*, 2013
* Papilio zelicaon* (Insecta)	Terrestrial	454	RNA-seq	x	x					Whole body (larvae)	Genetic reference	O'Neil *et al.*, 2010
* Tigriopus californicus* (Maxillopoda)	Marine	Illumina	RNA-seq	x				x	x	Whole body	Heat stress	Schoville *et al.*, 2012
Chordata												
*Ictalurus furcatus/Ictalurus punctatus* (Actinopterygii)	Freshwater	Illumina	RNA-seq	x				x	x	Gill, liver	Heat stress; common garden	Liu *et al.*, 2013
* Lates calcarifer* (Actinopterygii)	Marine	Illumina	RNA-seq	x				x	x	Muscle	Heat stress; common garden	Newton *et al.*, 2013
* Melanotaenia duboulayi* (Actinopterygii)	Freshwater	Illumina	RNA-seq	x				x	x	Liver	Heat stress	Smith *et al.*, 2013
* Ochotona princeps* (Mammalia)	Terrestrial	454	RNA-seq	x	x		x		x	Brain, gonad, heart, liver, lung	Observational	Lemay *et al.*, 2013
* Oncorhynchus mykiss* (Actinopterygii)	Freshwater	Illumina	RNA-seq	x				x	x	Gill	Common garden	Tan *et al.*, 2012
		Illumina	DNA(RAD)-seq		x		x		x	Liver	Observational	Narum *et al.*, 2013
* Pagothenia borchgrevinki* (Actinopterygii)	Marine	454, Illumina	RNA-seq	x						Gill, liver	Genetic reference	Bilyk and Cheng, 2013
* Paradoxornis webbianus* (Aves)	Terrestrial	Illumina	RNA-seq	x	x	x				Brain, liver	Genetic reference	Chu *et al.*, 2012
* Poecile atricapillus* (Aves)	Terrestrial	Illumina	RNA-seq	x				x	x	Hippocampi	Common garden	Pravosudov *et al.*, 2013
* Salmo salar* (Actinopterygii)	Marine	454	RNA-seq	x				x	x	Liver	Heat stress	Olsvik, *et al.*, 2013
* Trematomus bernacchii* (Actinopterygii)	Marine	454	RNA-seq	x				x	x	Brain, gill, Liver	Heat stress	Huth and Place, 2013
Cnidaria												
* Acropora millepora* (Cnidaria)	Marine	454	RNA-seq	x	x					Whole body (larvae)	Genetic reference	Meyer *et al.*, 2009
		SOLiD	RNA-seq					x	x	Whole body (larvae)	Heat stress	Meyer *et al.* 2011
* Acropora palmate* (Cnidaria)	Marine	454	RNA-seq	x	x	x	x		x	Whole body (larvae)	Observational	Polato *et al.*, 2011
* Porites astreoides* (Anthozoa)	Marine	454	RNA-seq	x				x	x	Skeleton	Heat stress	Kenkel *et al.*, 2013
Mollusca												
* Crassostrea gigas* (Bivalvia)	Marine	SOLiD	RNA-seq					x	x	Mantle	Heat stress	Clark *et al.*, 2013
* Haliotis rufescens* (Gastropoda)	Marine	Illumina	RNA-seq	x	x		x		x	Mantle	Observational	De Wit and Palumbi, 2013
* Macoma balthica* (Bivalvia)	Marine	454	RNA-seq	x	x		x		x	Whole body	Observational	Pante *et al.*, 2012
* Nucella lapillus* (Gastropoda)	Marine	Illumina	DNA(RAD)-seq; RNA-seq	x	x		x		x	Whole body	Observational	Chu *et al.*, 2014
* Villosa lienosa* (Bivalvia)	Freshwater	Illumina	RNA-seq	x				x	x	Mantle, muscle, gill	Heat stress	Wang *et al.*, 2012

Abbreviations: DGE, differential gene expression; GO, gene ontology; NGS, next-generation sequencing; SNP, single-nucleotide polymorphism.

**Table 2:** Recurrent GO terms emerging from intrapopulation DGE studies

*GO catergory*	*Recurrent GO terms (%)*
Biological processes	Translation (35.7%); metabolic processes (28.5%)
Molecular function	ATP binding (37%); structural constituent of ribosome (37%)
Cellular component	Ribosome (50%); membrane (37%)

Abbreviations: DGE, differential gene expression; GO, gene ontology.

Percentage of representation within studies is reported in parentheses.
