# Supplementary material for: The environmental genomics of metazoan thermal adaptation
Source: Heredity (Edinb). 2015 Mar 4;114(5):502–14. doi: 10.1038/hdy.2014.119 (PMC4815515; doi:10.1038/hdy.2014.119)
Supplement: Supplementary Information Methods [file hdy2014119x1.docx]

**Supplementary Information to:**

**The environmental genomics of metazoan thermal adaptation**

Damiano Porcelli^1^, Roger K. Butlin^1,2^, Kevin J. Gaston^3^, Dominique Joly^4^, and Rhonda R. Snook^1^

^1^Department of Animal and Plant Sciences, University of Sheffield, Sheffield, UK

^2^Sven Lovén Centre – Tjärnö, University of Gothenburg, Strömstad, Sweden.

^3^Environment and Sustainability Institute, University of Exeter, Penryn, UK

^4^Laboratoire Evolution, Génomes et Spéciation, CNRS – UPR 9034, Gif sur Yvette Cedex, France

Correspondence: D. Porcelli, Department of Animal and Plant Sciences, University of Sheffield, Sheffield, UK. Email: [d.porcelli@sheffield.ac.uk](mailto:d.porcelli@sheffield.ac.uk)

**Transcriptome assembly analysis.**

The construction of a high quality transcriptome is clearly critical for accurate downstream analyses.

In our survey, we found a high discrepancy in the mean number of reads and giga base pairs (Gp) generated per study, depending on the sequencing platform used: ~196M reads (~16Gb) for Illumina; ~1.3M reads (~0.4Gb) for Roche 454.

Short read sequencing platforms, such as Illumina, have the advantage of providing a very deep coverage, which confers greater accuracy in gene expression profiling tasks, and lowers the sequencing error rate (Loman *et al*., 2012).

Given recent bioinformatic improvements, a variety of assemblers can perform *de novo* transcriptome assembly utilising solely short read RNA-seq data (see for example Zhao *et al*., 2011). However, we observed that, when combined into transcripts, Illumina short reads generally resulted in oversized assemblies, comprising a much higher number of contigs (Mean = 178592, N = 11, 10 studies use paired-end reads, one study uses single-end reads) compared to transcriptomes deriving from Roche 454 reads (Mean = 53511, N = 9) or combinations of Roche 454 and Illumina reads (Mean = 64653, N = 3).

We identified a significant negative relationship between the number of assembled contigs within a transcriptome and the mean length of the reads used to generate it (p value = 0.00325, R^2^ = 0.3009, Supplementary Figure S1), while the factors “reads number” or “total number of base pairs sequenced” did not show any statistical correlation with the number of assembled contigs (p-values of 0.1722 and 0.413, respectively). Although we did not investigate this relationship further , it may depend on current technical limitations in resolving issues like complexity and higher levels of gene fragmentation emerging in short reads assemblies. For example, regions containing large tandem codon repeats or multi-modular repeats, such as for structural and assembly proteins, can lead to high complexity due to the generation of chimeric or truncated transcripts (for further considerations see Riesgo *et al*., 2012 and Klassen and Currie, 2012).

**Gene overlap between “Clinal” and “Experimental evolution” population genetic studies in *D. melanogaster*.**

Five genes were found to be in common between clinal outliers from natural *D. melanogaster* populations (N=807, Reinhardt *et al*., 2014, personal communication from the corresponding author) and lab strains subjected to experimental evolution in hot and cold environments (N=47, Tobler *et al*., 2014). *l(3)L1231* and *abnormal spindle* (*asp*) genes were found to map relatively close to *In(3R)Payne* inversion break points, while *CG6733* maps within the inversion itself (see their distribution on the *D. melanogaster* polytene Chromosome 3R map below). The other two genes were *decapentaplegic* (*dpp*, cytogenetic map 22F1-22F3, Chromosome 2L) and *retained* (*retn*, cytogenetic map 59F5, Chromosome 2R).


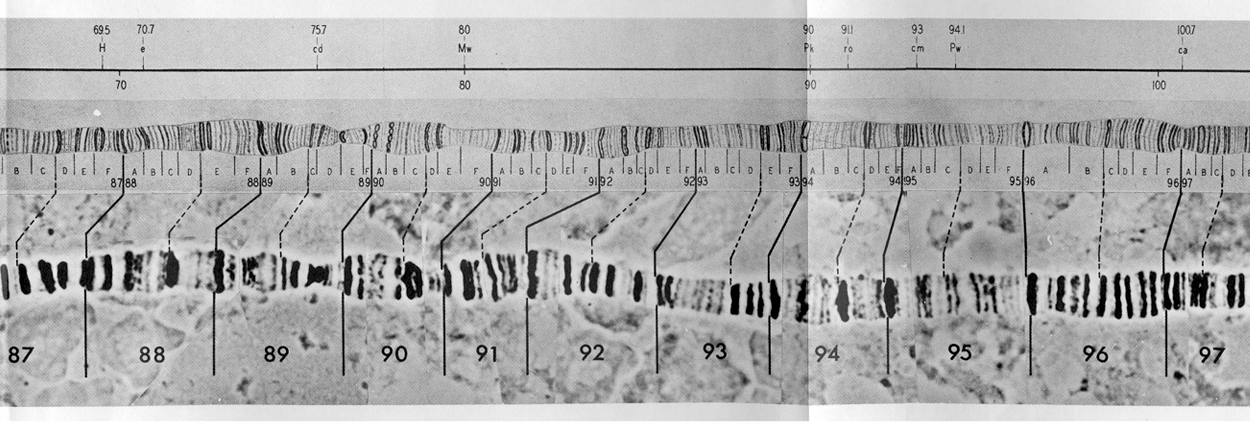


***CG6733***

***asp***

**Locus inverted in *In(3R)Payne***

***l(3)L1231***


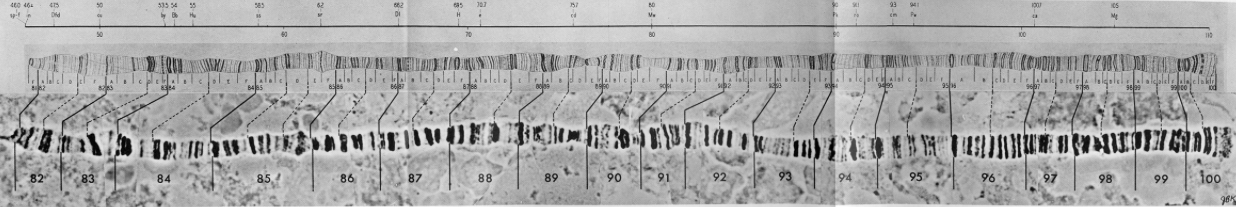


***D. melanogaster* Chr. 3R**

**Gene overlap between CESAR dataset and energy metabolism genes in *Drosophila melanogaster***

In order to detect a possible role of energy metabolism genes during stress responses in *D. melanogaster,* we tested the overlap between the curated CESAR dataset and genes involved in the oxidative phosphorylation and the Krebs cycle. 7 Oxphos genes were found to ovelap: *mtacp1, CG10219, CoVIIc, ND75, Cyt-c-p, Etf-QO, blw*; and 2 genes involved in the Krebs cycle: *CG10219* and *CG4095*.

**References**.

Klassen JL, Currie CR (2012). Gene fragmentation in bacterial draft genomes: extent, consequences and mitigation. *BMC Genomics* **13**: 14.

Loman NJ, Misra R V, Dallman TJ, Constantinidou C, Gharbia SE, Wain J, et al. (2012). Performance comparison of benchtop high-throughput sequencing platforms. *Nat. Biotechnol*. **30**: 434–9.

Reinhardt JA, Kolaczkowski B, Jones CD, Begun DJ, Kern AD (2014). Parallel geographic variation in Drosophila melanogaster. *Genetics* **197**: 361-73.

Riesgo A, Andrade SCS, Sharma PP, Novo M, Pérez-Porro AR, Vahtera V, et al. (2012). Comparative description of ten transcriptomes of newly sequenced invertebrates and efficiency estimation of genomic sampling in non-model taxa. *Front. Zool*. **9**: 33.

Tobler R, Franssen SU, Kofler R, Orozco-Terwengel P, Nolte V, Hermisson J, et al. (2014). Massive habitat-specific genomic response in D. melanogaster populations during experimental evolution in hot and cold environments. *Mol. Biol. Evol*. **31**: 364–75.

Zhao Q-Y, Wang Y, Kong Y-M, Luo D, Li X, Hao P (2011). Optimizing de novo transcriptome assembly from short-read RNA-Seq data: a comparative study. *BMC Bioinformatics* **12 Suppl 1**: S2.
