# Supplementary material for: The environmental genomics of metazoan thermal adaptation
Source: Heredity (Edinb). 2015 Mar 4;114(5):502–14. doi: 10.1038/hdy.2014.119 (PMC4815515; doi:10.1038/hdy.2014.119)
Supplement: Supplementary Table Legends [file hdy2014119x2.docx]

**Table S1**(provided as separate attachment). GO term analysis of the 1307 genes from the CESAR dataset. The analysis has been done using DAVID (Huang *et al.*, 2009).

**Table S2**(provided as separate attachment). GO term analysis of the 98 *D. melanogaster* shared genes between the 807 genes from the natural clinal study (Reinhardt *et al.*, 2014; personal communication from the corresponding author) and the full CESAR dataset. The analysis has been done using DAVID (Huang *et al.*, 2009).

**Table S3**(provided as separate attachment). Number of differentially expressed transcripts (DET) and relative experimental conditions for differential gene expression (DGE) studies.

**Figure S1.** Main classes of gene function within the CESAR dataset. Each category presents a degree of overlap with others due to the presence of multifunctional genes, as shown by the Venn diagram.

**Figure S2**. Statistical relationship between the number of assembled contigs and read length within transcriptome assemblies from the reviewed literature.

**Figure S3**. Correlation between the number of differentially expressed genes and the duration of the temperature shift within intra-population DGE studies. **a**) tissue specific analysis; **b**) all data analysed, including multiple data points from the same species; **c)** data excludes the three studies on *Trematomus bernacchii* which exhibited the highest number of differentially expressed genes, as seen in Fig. S3b.
