# Supplementary material for: The environmental genomics of metazoan thermal adaptation
Source: Heredity (Edinb). 2015 Mar 4;114(5):502–14. doi: 10.1038/hdy.2014.119 (PMC4815515; doi:10.1038/hdy.2014.119)

**Figure S1.** Main classes of gene function within the CESAR dataset. Each category presents a degree of overlap with others due to the presence of multifunctional genes, as shown by the Venn diagram.

**
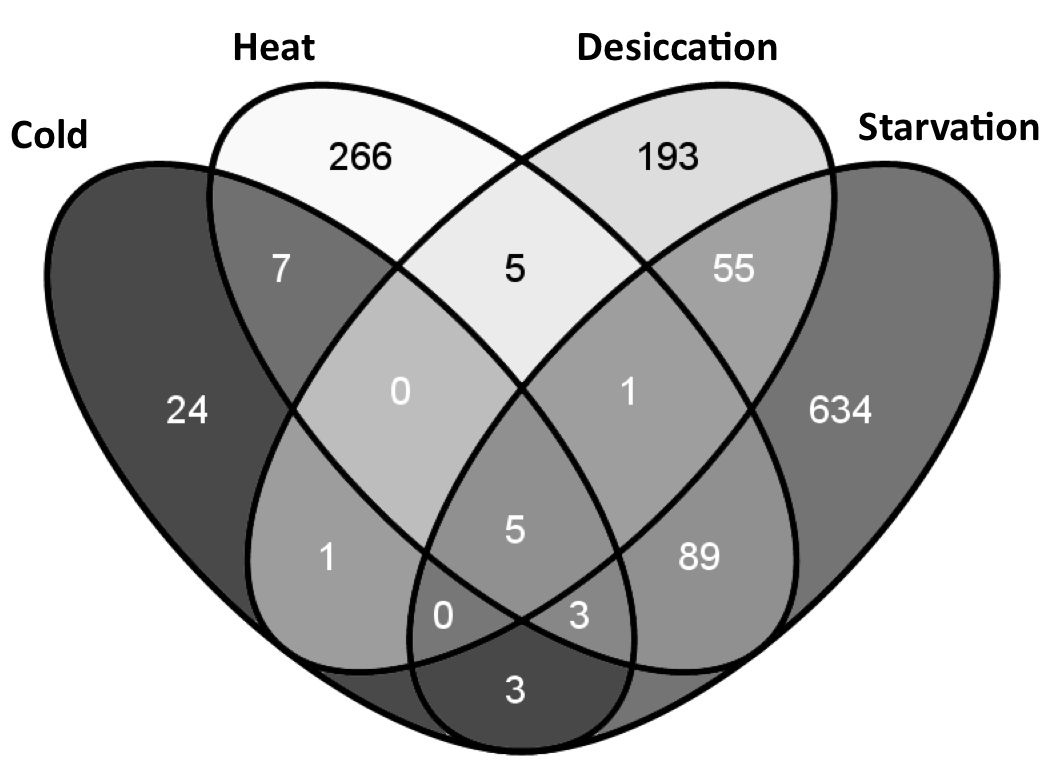
**

**Figure S2**. Statistical relationship between the number of assembled contigs and read length within transcriptome assemblies from the reviewed literature.

**
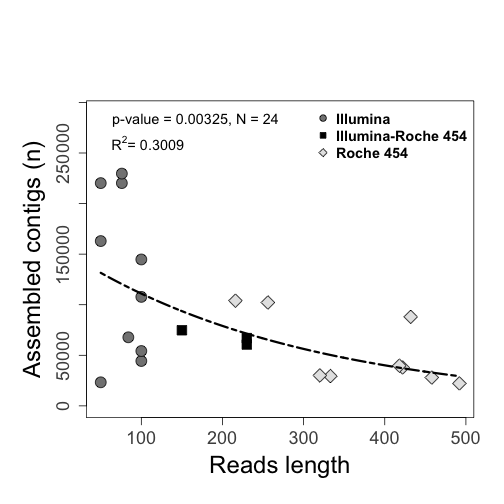
**

**Supplementary Figure S3**. Correlation between the number of differentially expressed genes and the duration of the temperature shift within intra-population DGE studies. **a**) tissue specific analysis; **b**) all data analysed, including multiple data points from the same species; **c)** data excludes the three studies on *Trematomus bernacchii* which exhibited the highest number of differentially expressed genes, as seen in Fig. S3b.

**a b c**


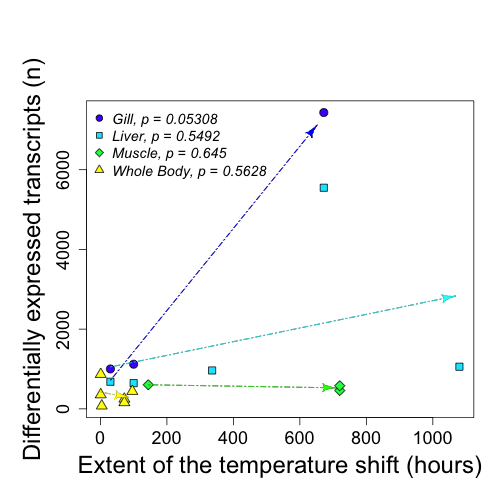

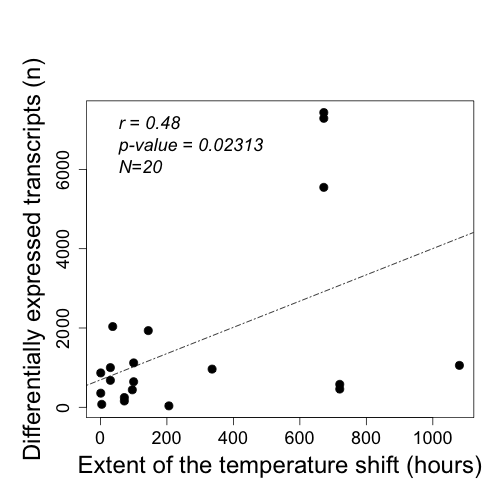

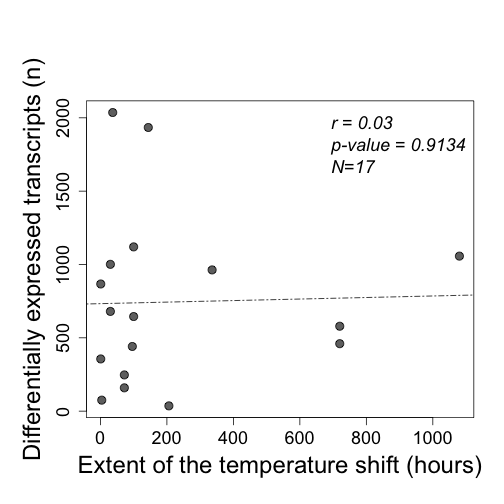

Supplement: Supplementary Figures [file hdy2014119x3.docx]
